# Aldosterone Synthase Inhibitors and Dietary Interventions: A Combined Novel Approach for Prevention and Treatment of Cardiovascular Disease

**DOI:** 10.7759/cureus.36184

**Published:** 2023-03-15

**Authors:** Ayoola Awosika, Anosh Khan, Uzochukwu Adabanya, Adekunle E Omole, Richard M Millis

**Affiliations:** 1 College of Medicine, University of Illinois Chicago, Chicago, USA; 2 Internal Medicine, Spartan Health Sciences University School of Medicine, Vieux Fort, LCA; 3 Community Medicine, Mercer University School of Medicine, Columbus, USA; 4 Anatomical Sciences, American University of Antigua College of Medicine, Coolidge, ATG; 5 Pathophysiology, American University of Antigua College of Medicine, Coolidge, ATG

**Keywords:** aldosterone synthase inhibitor, systemic hypertension, dash diet, baxdrostat, treatment-resistant hypertension, renin–angiotensin–aldosterone blockade

## Abstract

Systemic hypertension (HTN) is the hallmark of cardiovascular disease and the forerunner of heart failure. These associations have been established over decades of research on essential HTN. Advancements in the treatment of patients diagnosed with HTN, consisting of alpha- or beta-adrenergic receptor blockers, calcium channel blockers, angiotensin-converting enzyme inhibitors, thiazide, or aldosterone receptor blockers known as anti-mineralocorticoids, in the presence or absence of low sodium salt diets, often fail to control blood pressure adequately to prevent morbidity and mortality. Low sodium diets have had limited success in controlling HTN because low sodium intake is associated with renin-angiotensin-aldosterone system upregulation. Therefore, upregulating aldosterone secretion, sodium, and water retention which, in turn, moves the blood pressure back toward the range of HTN dictated by the baroreceptor reset value, as a compensatory mechanism, especially in resistant HTN. These impediments to blood pressure control in HTN may have been effectively circumvented by the advent of a new class of drugs known as aldosterone synthase inhibitors, represented by baxdrostat. The mechanism of action of baxdrostat as an aldosterone synthase inhibitor demonstrates the inextricable linkage between sodium and blood pressure regulation. Theoretically, combining a low sodium diet with the activity of this aldosterone synthesis inhibitor should alleviate the adverse effect of renin-angiotensin-aldosterone system upregulation. Aldosterone synthesis inhibition should also decrease the oxidative stress and endothelial dysfunction associated with HTN, causing more endothelial nitric oxide synthesis, release, and vasorelaxation. To the best of our knowledge, this is the first systematic review to summarize evidence-based articles relevant to the use of a novel drug (aldosterone synthase inhibitor) in the treatment of HTN and cardiovascular disease. Making the current database of relevant information on baxdrostat and other aldosterone synthase inhibitors readily available will, no doubt, aid physicians and other medical practitioners in their decision-making about employing aldosterone synthase inhibitors in the treatment of patients.

## Introduction and background

Cardiovascular disease (CVD) is the number one cause of mortality among American adults [1]. To reduce the chance of experiencing recurrent cardiovascular events, patients with established CVD have challenges in attaining cardiovascular risk reduction. Such reduction of cardiovascular risk can best be accomplished by adhering to recommended pharmacotherapy that favorably alters the main coronary risk factors (i.e., hypertension (HTN), diabetes, and hypercholesterolemia). The most common modifiable CVD risk factor, affecting almost one in three patients, is HTN [1]. It is, therefore, imperative to control HTN optimally to reduce its long-term consequences, such as heart failure, atrial fibrillation, peripheral vascular disease, aortic syndromes, and chronic kidney disease (CKD).

The renin-angiotensin-aldosterone system (RAAS) produces the end-product aldosterone. Aldosterone functions principally as a mineralocorticoid hormone that regulates blood plasma fluid and electrolyte levels. The development of systemic HTN, as well as the changes brought on by HTN to the heart, kidneys, and peripheral vasculature, are influenced by a higher plasma level of aldosterone. Inhibition of the effect of aldosterone is, therefore, an essential goal for the treatment of CVD [1]. There are currently two mineralocorticoid receptor antagonists (MRAs) that target aldosterone in the treatment of HTN, spironolactone, and eplerenone. MRAs lower blood pressure (BP), particularly in resistant HTN, and improve congestive heart failure (CHF) outcomes by preventing aldosterone from adhering to its receptor. Spironolactone is known to have some adverse side effects on the endocrine system that produces significant limitations for its use. Because aldosterone is the principal regulator of plasma potassium by stimulating the excretion of excess potassium, the use of spironolactone is linked to hyperkalemia and compensatory increases in plasma aldosterone levels [2]. This compensatory increase in the secretion of aldosterone may, therefore, exacerbate effects of aldosterone that are mediated independently of its effects on gene transcription, or what are known as "nongenomic effects" [2-4]. These impediments to the usage of MRAs appear to have been circumvented by inhibiting the production of aldosterone. Aldosterone synthase inhibitors are, therefore, a novel strategy for reducing the amount of aldosterone to which the cardiovascular system is exposed [5].

Sodium intake and HTN

The links between high dietary sodium intake and elevation of BP are elucidated and described in virtually all textbooks of medical physiology. Guyton’s textbooks of medical physiology explain the linkage between sodium intake and BP by demonstrating that high dietary salt intake raises plasma osmolarity which, in turn, induces secretion of arginine-vasopressin (AVP), also known as anti-diuretic hormone (ADH) [6]. AVP/ADH increases the reabsorption of water primarily from the collecting ducts of the kidney, which decreases free water clearance and increases the retention of plasma water. Plasma water retention increases the circulating blood volume, cardiac output, renal perfusion pressure, glomerular filtration rate, and sodium excretion [6,7], as the body attempts to restore normal plasma sodium and circulating blood volume.

HTN appears to result from an abnormality in the ability of the kidney to excrete excess dietary sodium, thereby retaining the sodium and water in the blood plasma [8]. Sodium and water retention can also occur whenever RAAS function deteriorates [9], the same as what happens when AVP/ADH is secreted in response to the high plasma osmolarity. It is noteworthy that the normal process of aging decreases the number of functional nephrons, leading to sodium and water retention, as well as elevated BP. The increased BP associated with aging may be thought of as a compensatory mechanism to decrease the plasma sodium load by pressure natriuresis [10].

We, therefore, designed this review to explore all evidence-based articles that pertain to the use of this novel drug (aldosterone synthase inhibitor) in the treatment of cardiovascular disease with special emphasis on systemic and resistant HTN. This will, no doubt, bring a paradigm shift to aid physicians and other medical practitioners in their decision-making about employing this novel strategy in the treatment of HTN.

## Review

Materials and methods

For this systematic review, several scientific databases, listed in Table 1, were searched for randomized controlled clinical trials (RCTs) that have performed experiments involving the novel class of drugs known as aldosterone synthase inhibitors (ASIs) from database inception up until January 2023. We deployed database-specific search keywords to include aldosterone synthase inhibitor, LCI699, LY3045697, baxdrostat, hypertension, and resistant hypertension. Then, using the Preferred Reporting Items for Systematic reviews and Meta-Analyses (PRISMA) Statement 2020 guidelines, the titles and abstracts were reviewed. This was followed by a full manuscript review by two authors, with a third party for any disagreements. Additionally, an umbrella search of shortlisted studies was conducted, and the reference lists were scanned for any additional relevant RCTs. Detailed search criteria guided by the patient/population, intervention, comparison, and outcomes (PICO) strategies are described in Table 1.

**Table 1:** Search strategy and criteria A detailed description of PubMed, Scopus, Google Scholar, Cochrane library, EBSCO, MEDLINE, and EMBASE scientific literature database searches employing PICO criteria for evidence-based medicine investigations. MeSH: Medical subject headings; EBSCO: Elton B. Stephens Company database; MEDLINE: Medical Literature Analysis and Retrieval System Online; EMBASE: Excerpta Medica Database; PICO: patient/population, intervention, comparison, and outcome

MeSH terms used to guide search strategy	Population: Hypertensive patients (essential hypertension, or resistant hypertension)
	Intervention: Aldosterone synthase inhibitor treatment, LCI699 or LY3045697 or baxdrostat
	Comparison: No treatment (placebo)
	Outcome: Reduction in systolic and diastolic blood pressure, reduction of aldosterone or cortisol
Sources of evidence searched	PubMed, Scopus, Google Scholar, Cochrane library, EBSCO, MEDLINE, EMBASE
Inclusion criteria	All studies published (no limitation by publication year)
	No language restriction
	Randomized control trial and clinical trial
	Studies pertaining to aldosterone synthase inhibitor use an anti-hypertensive
Exclusion criteria	Exclusion of duplicates, editorials, and reviews
	Article title and abstract that does not focus on aldosterone synthase inhibitor treatment of hypertension

Results of best evidence

**Figure 1:**
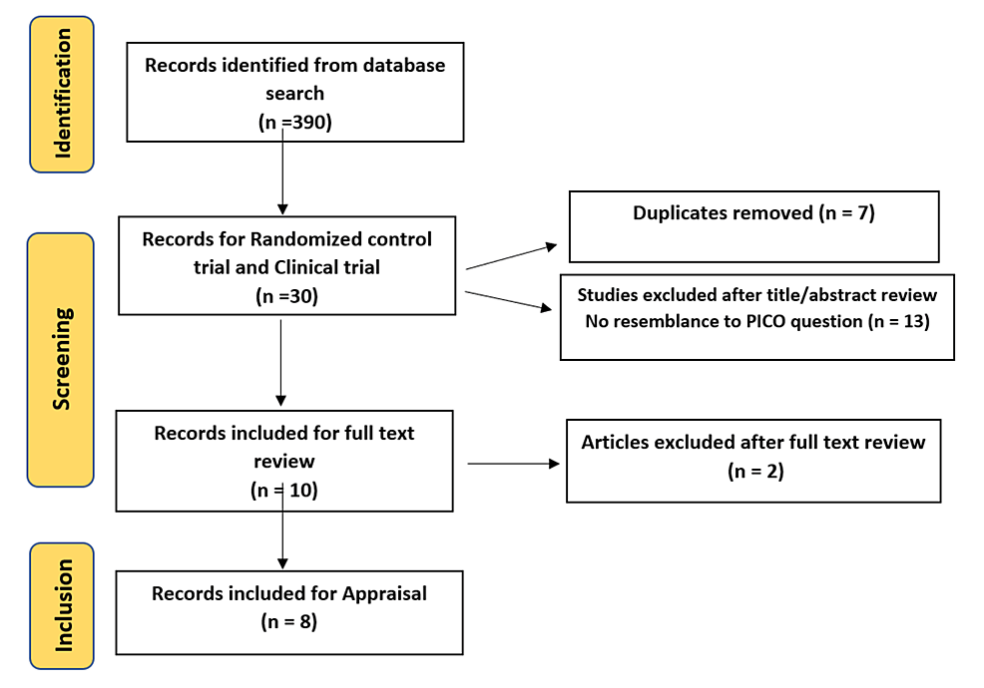
Results of literature search.

Eight articles were found to be highly relevant to this systematic review based on the inclusion criteria, shown in the search results depicted in Figure 1. The articles were further assessed based on the critical appraisal skills program (CASP) scale for RCTs, a scale that measures the methodological quality of RCTs.

Table 2 compares the key characteristics of each of the eight highly relevant articles identified by the search. The tabulation is presented in reverse chronological order, for convenience, to facilitate reading and comparisons.

**Table 2:** Aldosterone synthase inhibitor studies HTN: hypertension; SBP: systolic blood pressure; DBP: diastolic blood pressure; QD: once per day; BID: twice per day; Tx: treatment; ACTH: adrenocorticotrophic hormone; AE: adverse event; CASP: critical appraisal skills program; ASI: aldosterone synthase inhibitor

Citation	Study design	Population	Intervention	Outcome measures	Results	Adverse events	Recommendation	Level of evidence	Quality assessment score (CASP)
Freeman et al., 2023 [11]	Phase 1 prospective RCT	Healthy volunteers (N= 56)	Baxdrostat QD for 10 days at varying doses (0.5 mg, 1.5 mg, 2.5 mg, or 5 mg vs. placebo, also on low salt vs. normal salt diet	Ascending doses of baxdrostat evaluated for efficacy, safety, pharmacodynamics, and pharmacokinetics	Baxdrostat had rapid absorption, with peak plasma concentration seen at 4 hours post administration, mean half-life of 26-31 hours, with biphasic decline; dose-dependent decrease in aldosterone (51-73% on day 10) at doses ≥ 1.5 mg, regardless of low or normal salt diet; increase in 11-deoxycorticosterone; no effect on cortisol with ACTH challenge in groups on low salt diet	No serious adverse event or death	Drug was well tolerated and safe; selectively inhibits aldosterone synthase without cortisol interference	1b	10
Freeman et al., 2022 [12]	Multicenter phase 2 prospective RCT	Patients with resistant HTN having taken at least three different anti-hypertensive drugs (N=274)	Baxdrostat QD vs, placebo for 12 wks, 0.5, mg, 1 mg, or 2 mg	Differences in BP for each baxdrostat treatment group vs. placebo	Baxdrostat produced dose-dependent decrease in aldosterone and systolic BP (−20.3 mmHg, −17.5 mmHg, −12.1 mmHg, and −9.4 mmHg in the 2 mg, 1 mg, 0.5 mg, and placebo groups, respectively); the 2 mg dose decreased diastolic BP by 14.3 mmHg; plasma renin increased by 13.8 ng/ml per h at the 2 mg dose; no cortisol effect.	No serious adverse event or death	Drug produced a dose-related decrease in BP; safe and effective in resistant HTN	1b	10
Lancaster et al., 2017 [13]	Multiple prospective RCT	Healthy volunteers (N=51)	Randomization to LY3045697 vs. placebo or 25mg spironolactone	Tolerability, safety, efficacy, BP, plasma aldosterone, cortisol, renal Na+, and K+ excretion	Peak plasma concentration within 0.5 h, half-life 10 h, biphasic decline; dose-dependent decrease in aldosterone after 4 hours of 1 mg LY3045697, larger doses resulted in near complete suppression of aldosterone; no evidence of nephro- or hepatotoxicity; with no change in cortisol	No serious adverse event or death	Effective and potent inhibitor of aldosterone, cortisol-sparing; spironolactone-like, anti-androgenic effects absent	1b	9
Menard et al., 2014 [14]	Multicenter prospective RCT	Healthy male volunteers (N= 99)	Single dose phase: 37 subjects given ASI LCI699 in single dose phase vs. placebo Multiple-dose phase: 62 subjects given ASI LCI699 vs. placebo	Safety, pharmacokinetics, and pharmacodynamics	LCI699 showed maximum plasma level in 1 h; half-life 3.4-4.8 h; aldosterone decreased by 39% (urine), 49% (plasma); deoxycorticosterone and urinary potassium excretion increased, with increase in renin and natriuresis; doses >1mg inhibited basal and ACTH-stimulated cortisol	Headache and dizziness without hypotension common, not dose-dependent.	Selective inhibition of aldosterone synthase increases renin and decreases cortisol	1b	9
Schumacher et al., 2013 [15]	Multicenter phase 2 prospective RCT	Patients with essential HTN, resistant/ uncontrolled HTN, and primary hyperaldosteronism	LCI699 4 Tx groups: 1. Primary hyperaldosteronism 4 weeks Tx (n-14); 2. Essential HTN vs. placebo Tx 8 weeks; 3. Essential HTN (on at least 1 anti-HTN drug Tx 6 weeks; 4. Resistant HTN 8 weeks Tx	LC1699 efficacy, safety	Essential HTN group had reduction in both SBP and DBP vs. placebo Hyperaldosteronism group had reduction in SBP Resistant HTN group BP reduction on LCI699 inferior to on eplerenone (LCI699 0.25 mg BID –11.4 mmHg; 0.5/1.0 mg BID –12.5 mmHg; 1.0 mg QD – 13.1 mmHg; eplerenone 50 mg BID – 18.7 mmHg; versus placebo - 8.8 mmHg	No serious adverse event or death was reported.	LCI699 treatment resulted in a dose-dependent and time-dependent (frequency) reduction in ambulatory systolic and diastolic BP. Also dose-dependence of the ACTH stress.	1b	9
Karns et al., 2013 [16]	Multicenter phase 2 prospective RCT	Patients with resistant hypertension (N= 155)	Control period 2 weeks Tx single-blind placebo followed by randomization to 5 groups to receive LCI699 0.25 mg BID) or 1 mg QD or 0.5 mg BID titrated to 1 mg BID after 4 weeks or to 50 mg eplerenone BID or placebo Tx 8 weeks	LC 1699 efficacy, safety	LCI699 produced dose-dependent reduction in plasma aldosterone while plasma renin and plasma 11-deoxycorticosterone increased Reduction in resting SBP vs. placebo (NS) Largest placebo-adjusted reduction in ambulatory BP after 8 weeks in eplerenone group (SBP- 14.7 mmHg, DBP - 9.4 mmHg; P< 0.001)	Only one serious AE in the eplerenone group	LCI699 treatment as an aldosterone synthase inhibitor demonstrated more BP lowering efficacy in patients with essential HTN than resistant HTN.	1b	10
Andersen et al., 2012 [17]	Multicenter phase 2 prospective RCT	Patients with essential HTN achieving no BP control despite one or more anti-hypertensive therapy (N= 63)	Randomization to 5 Tx groups: LCI699 0.5mg QD, 1 mg QD 1 mg BID, 2 mg QD or placebo 6 weeks Tx	LC1699 efficacy, safety, pharmaco- kinetics, dynamics, ACTH-stimulated cortisol	LCI699 peak plasma concentration within 1 h indicates rapid oral absorption, with half-life between 3.8-5.5 hours; All 4 groups had reduction in sitting SBP and DBP, with only 1 mg QD, and 2 mg QD vs. placebo -9.1 mmHg, P=0.09; -9.2mmHg, P=0.007 Maximum tolerated dose ranges 0.88-1.81 mg QD, at 90% prediction interval	No serious AE. Most reported side effects were headache and dizziness	There is potentially dose-dependent and time-dependent (frequency) suppressive effect of LCI699 on both cortisol (ACTH-stimulated) and aldosterone pathway.	1b	9
Calhoun et al., 2011 [5]	Multicenter phase 2 prospective RCT	Patients with essential HTN (stage 1 to 2) treated with ≤2 anti-HTN drug or untreated (N= 524)	Randomization to 6 groups: LCI699 0.25 mg QD, 0.5mg QD, 1 mg QD, 0.5mg BID, eplerenone 50 mg or placebo for 8 weeks Tx	LC1699 efficacy, safety, tolerability	LC1699 at all doses lowered 24-h SBP and DBP (P<0.01) 1 mg QD dose more effective than placebo at reducing DBP (P=0.001) and similar reduction with 50 mg BID dose eplerenone LCI699 reduced aldosterone while eplerenone increased aldosterone Both LCI699 and eplerenone increased renin; no change in AM cortisol for all Tx groups	Most frequently reported were headaches, dizziness, and nasopharyngitis.	LCI699 shows superior effect on SBP and DBP at higher doses than eplerenone. Overall, all doses of LCI699 lowered 24-hour and clinic BP.	1b	10

Critical appraisal of studies: implications for practice, education, and future research

The *CYP11B2* gene encodes aldosterone synthase (AS) and is strongly expressed in the glomerulosa cells of the adrenal gland. *CYP11B2* is expressed at lower levels in other organs [18,19]. AS sequentially undergoes 11-hydroxylation, 18-hydroxylation, and 18-oxidation to transform 11-deoxycorticosterone (11-DOC) into aldosterone [20, 21]. Selective inhibition of AS is shown to reduce aldosterone synthesis and mitigate its negative effects on both receptor-mediated and non-genomic processes [14]. This review identifies eight relevant studies that examined AS inhibitors (ASIs) and their effects on healthy volunteers, patients with essential HTN, resistant HTN, and individuals with hyperaldosteronism-related disorders.

The Calhoun et al. (2011) study tested LCI699, the first inhibitor of the enzyme AS, in patients diagnosed with stage 1-2 stage HTN who were either untreated or receiving two antihypertensive medications over an eight-week period [5]. The LCI699 1.0 mg one daily dose reduced resting, seated diastolic BP (DBP) more significantly than the placebo (P = 0.001). A similar decrease in DBP was found with eplerenone (50 mg twice daily). At eight weeks, all LCI699 doses reduced systolic BP (SBP) compared to the placebo (P<0.001). Epleronone produced a similar reduction in SBP at a 50 mg twice dose. In contrast to eplerenone, which increased aldosterone levels, LCI699 at a dose of 0.5 mg twice daily markedly decreased aldosterone 12 hours after the last dose. The once daily doses of both eplerenone and LCI699 increased plasma renin activity (PRA) compared to the placebo treatment, although the increase was not statistically significant. In summary, the Calhoun et al. study found that LCI699 was well-tolerated and effectively reduced BP compared to a placebo. However, they also found that a 1.0 mg once daily dose of LCI699 had similar effects to the 50 mg twice daily dose of eplerenone. The most significant finding of this trial is that LCI699 lowered plasma aldosterone, which could be beneficial for individuals at high risk for conditions related to excessive aldosterone, such as chronic renal disease, resistant HTN, and congestive heart failure.

The Andersen et al. (2012) study of patients with essential HTN who weren't responding to one or more antihypertensive drugs evaluated the safety and efficacy of LCI699 [17]. LCI699 decreased the adrenocorticotropic hormone (ACTH)-induced cortisol response in a dose- and time-dependent manner, with the impact becoming visible by day seven of the treatment. The exposure-response analysis estimated the mean tolerable dose (MTD) to be 1.30 mg once daily with a 90% prediction range of 0.88-1.81 mg once daily based on the protocol-defined MTD of 20% of patients having a 400 nmol/L plasma cortisol level after ACTH stimulation. Despite the LC1699 suppression of the ACTH-cortisol axis, none of the study subjects displayed symptoms or indicators of adrenal insufficiency. In summary, the Andersen et al. (2012) study found that LCI699 has limited target selectivity as well as a negative impact on the production of cortisol. The cortisol-reducing effect of LC1699 may be an effective treatment for HTN alone or HTN associated with aldosterone and cortisol-induced cardiomyopathies where smaller amounts of the drug are needed or when inhibition of both aldosterone and cortisol synthesis could be beneficial.

The Karns et al. (2013) study involved giving LCI699 to patients with resistant HTN who weren't able to control their BP on a consistent regimen of three or more medications, including a diuretic, for at least four weeks [16]. All LCI699 groups experienced minor decrements in mean seated SBP (MSSBP) after eight weeks; however, none of these decrements were statistically different from the placebo. Placebo-adjusted MSSBP reductions with LCI699 1 mg once daily and 0.5 or 1.0 mg twice daily were quantitatively greater (4.3 mmHg), but the largest placebo-adjusted MSSBP reduction was seen in the group given eplerenone 50 mg twice daily (9.9 mmHg, P= 0.017 vs. placebo). In terms of biomarkers, LCI699 suppressed (decreased) plasma aldosterone and increased PRA and 11-deoxycorticosterone in a dose-dependent manner compared to the placebo. However, eplerenone failed to suppress and was found to increase both plasma aldosterone and PRA. The Karns et al. (2013) study demonstrates that although LCI699 caused minor reductions in BP in patients with resistant HTN, the decrements in BP were not as large or significant as those caused by eplerenone [16]. Other researchers report significant decrements in SBP and DBP when using MRAs as a fourth-line treatment for resistant HTN [22,23]. It remains unclear why LCI699 performed less well than eplerenone in this trial and for what reasons. Higher dosages of LCI699 might be required to produce outcomes comparable to those of eplerenone. Higher doses of LC1699 than those currently employed are not likely to be useful because of the potential adverse effects of LC1699 on cortisol synthesis [5,14,17].

The Schumacher et al. (2013) study investigated the safety and efficacy of LCI699 in hypertensive patients [15]. The findings demonstrated that the once daily and twice daily regimens of LCI699 effectively decreased plasma aldosterone levels and reduced both SBP and DBP in patients with essential HTN, although these benefits were not as pronounced in patients with primary hyperaldosteronism or resistant HTN. Patients with primary hyperaldosteronism had marginally lower 24-hour and daytime ambulatory SBP than those with essential HTN. A significant decrement in BP was not seen in this study despite the effective treatment of hypokalemia associated with high plasma aldosterone levels. LCI699 had less of an impact than eplerenone on BP in patients with resistant HTN. At doses of 0.25 mg twice daily, 0.5 or 1.0 mg twice daily, and 1.0 mg once daily, the MSSBP reductions from baseline for LCI699 were -11.4 mmHg, -12.5 mmHg, and -13.1 mmHg, respectively. In contrast, the MSSBP reduction from baseline for eplerenone at a dose of 50 mg twice was -18.7 mm Hg, while it was -8.8 mmHg for the placebo group. The Schumacher et al. (2013) study demonstrates that LCI699 was useful for lowering BP in patients with essential HTN, primary hyperaldosteronism or resistant HTN [15]. Given that high doses of spironolactone have been linked to endocrine side effects, LCI699 appears to produce better control of aldosterone and potassium and may therefore be a substitute or adjunct to spironolactone for conditions, such as primary hyperaldosteronism or liver cirrhosis.

The Menard et al. (2014) study tested LCI699 on healthy male subjects [14]. The levels of plasma and urine aldosterone were reduced by LCI699 single doses (3 and 200 mg) by 60-78% and 68-81%, respectively. There were significant decrements in urine cortisol at doses of 30 mg, 100 mg, or 200 mg LC1699. At low doses, LCI699 appears to increase 11-deoxycorticosterone by targeting the 11-hydroxylase enzyme. Plasma 11-deoxycorticosterone increased at LC1699 doses of 100 mg (+942%) and 200 mg (+584%) compared to placebo (P< 0.001). On the first, seventh, and 14th day of a multiple-dose trial, LCI699 lowered plasma aldosterone by 49%, 47%, and 63%, respectively, for the 0.5 mg, 1 mg, and 3 mg dosages, all of which were found to be statistically significant compared to the placebo. The 0.5-3 mg dosages of LCI699 did not show any notable impact on 24-hour urine cortisol. The 24-hour urine cortisol level of one subject who received 10 mg of LCI699 on day seven did, however, decrease by more than 70%. An increase of 228% was observed in 11-deoxycorticosterone from the starting point when 3 mg of LCI699 was administered compared to placebo (P< 0.001); however, no change was seen after 0.5 mg or 1 mg of LC1699. On day six, the decrease in cortisol levels that were triggered by ACTH was not significantly affected by lower doses of the drug (0.5 mg, 1.8% reduction, and 1 mg, 11% reduction), despite a peak reduction of 22% from the initial levels (P<0.05 vs. placebo). On days six and 10, LCI699 (10 mg) further decreased ACTH-stimulated cortisol compared to the placebo (P< 0.05), and this effect vanished four to nine days after LCI699 administration was stopped. On the initial day of treatment, LCI699 generated a fast natriuresis, but by the seventh day, it had reached steady-state sodium (Na+) balance. On days one, seven, and 14, LCI699 caused modest increases in plasma K+ (average, +0.29 mEq/L) and urine K+ excretion (+37.6 mEq/24 h). Every dose of LCI699 significantly increased PRA. In summary, the Menard et al. (2014) study demonstrates that LCI699 is well tolerated by healthy volunteers; headaches, dizziness, and fatigue were the most frequent side effects [14].

In the Lancaster et al. (2017) trial, the ASI LY3045697 was studied in healthy volunteers [13]. LY3045697 has a 39-fold lower targeting of 11-hydroxylase activity than LC1699. Following a single dose of LY3045697, a dose-dependent decrease in urine aldosterone excretion was observed, starting at 1 mg and reaching statistical significance at 3 mg. LY3045697 inhibited 11-hydroxylase, which decreased aldosterone synthesis and increased levels of the aldosterone precursors corticosterone and 11-deoxycorticosterone. The broad range of LY3045697 dosages with high aldosterone synthesis inhibition carries a low risk of reducing cortisol production. This level of selectivity is significantly higher than that of LCI699. Serum potassium did not change after LY3045697 was administered once but after repeated administration, there was a dose-related increase in serum potassium levels over time. Comparable to 25 mg of spironolactone, LY3045697 had an impact on serum potassium at 1 mg, with greater effects shown at 10 mg and 100 mg. In summary, the Lancaster et al. (2017) study demonstrates that LY3045697 was well tolerated across the tested dose range, and no fatalities or severe adverse events were noted during the trial. LY3045697 exhibited significant aldosterone synthesis inhibition with greater cortisol-sparing activity than LCI699 [13].

The Freeman et al. (2023) study in healthy subjects shows the impact of a normal or low-salt diet on baxdrostat given once daily for 10 days [11]. Baxdrostat decreased plasma aldosterone at doses above 1.5 mg, and this effect was not affected by dietary salt intake. Under both normal and low-salt diet conditions, the effect of baxdrostat on plasma 11-deoxycorticosterone and cortisol was negligible, even when an ACTH challenge was added to the low-salt diet. Subjects on a low-salt diet exhibited elevation of plasma ACTH initially but were not significantly different from those given a placebo, and the increased ACTH was not significantly different from the low-salt diet and baxdrostat treatments. It is noteworthy that baxdrostat produced a dose-dependent reduction in ACTH in subjects on a normal salt diet. As expected, based on the decrease in aldosterone, baxdrostat produced moderate changes in serum sodium and potassium, with the degree of change varying depending on the dose administered. The Freeman et al. (2023) trial concluded that baxdrostat is safe because there were no fatalities, severe adverse events, or treatment terminations owing to treatment-emergent adverse events. These findings support the preclinical hypothesis that baxdrostat would specifically target aldosterone synthase, shown at doses of 1.5 mg and higher. Baxdrostat decreased plasma aldosterone in a dose-dependent manner and had no significant impact on plasma cortisol. The aldosterone-lowering effect of baxdrostat is reported to have persisted for the duration of the study, indicating the absence of compensatory mechanisms which distinguish baxdrostat from LCI699 and LY3045697.

Another Freeman et al. (2022) study evaluated baxdrostat in patients with treatment-resistant HTN [12]. Baxdrostat is shown to selectively inhibit AS without affecting 11-beta-hydroxylase [24]. The trial was discontinued early at 12 weeks due to its effectiveness criteria being met. The results showed that baxdrostat was associated with dose-dependent changes in SBP of -20.3 mmHg, -17.5 mmHg, and -12.1 mmHg at 2 mg, 1 mg, and 0.5 mg doses, respectively [12]; in comparison to -9.4 mmHg for the placebo. In contrast to the placebo, baxdrostat at the 1 mg and 2 mg doses significantly reduced MSSBP, whereas baxdrostat at 2 mg significantly reduced mean seated DBP. There were no serious side effects or instances of either hyperkalemia or adrenocortical insufficiency. The prevention and treatment of HTN with algorithm-based therapy-2 study and its sub-studies suggest that treatment-resistant HTN may be caused by autonomous aldosterone production, which may explain why an MRA (spironolactone) was found to be more effective in reducing BP than other anti-hypertensive drugs [25,26]. This trial adds to the growing evidence that aldosterone plays a key role in treatment resistance and that baxdrostat has the capacity to decrease both BP and aldosterone secretion [12]. In comparison to other HTN treatments, summarized in a study of 11,000 individuals from 42 different trials, it was discovered that patients who received angiotensin-converting enzyme inhibitors, calcium-channel blockers, or diuretics, in addition to their existing treatments, experienced an average decrease in SBP of 7-8 mmHg, compared to a placebo. In that regard, the groups given baxdrostat at doses of 2 mg (-11.0 mmHg) and 1 mg (-8.1 mmHg) had even greater reductions in SBP compared to the placebo (-9.4 mmHg, (P< 0.001 and P< 0.003, respectively). Without any change in plasma cortisol, these decrements in BP were associated with increased PRA and decreased plasma aldosterone [12].

Roles of aldosterone and aldosterone synthesis inhibition in HTN

**Figure 2:**
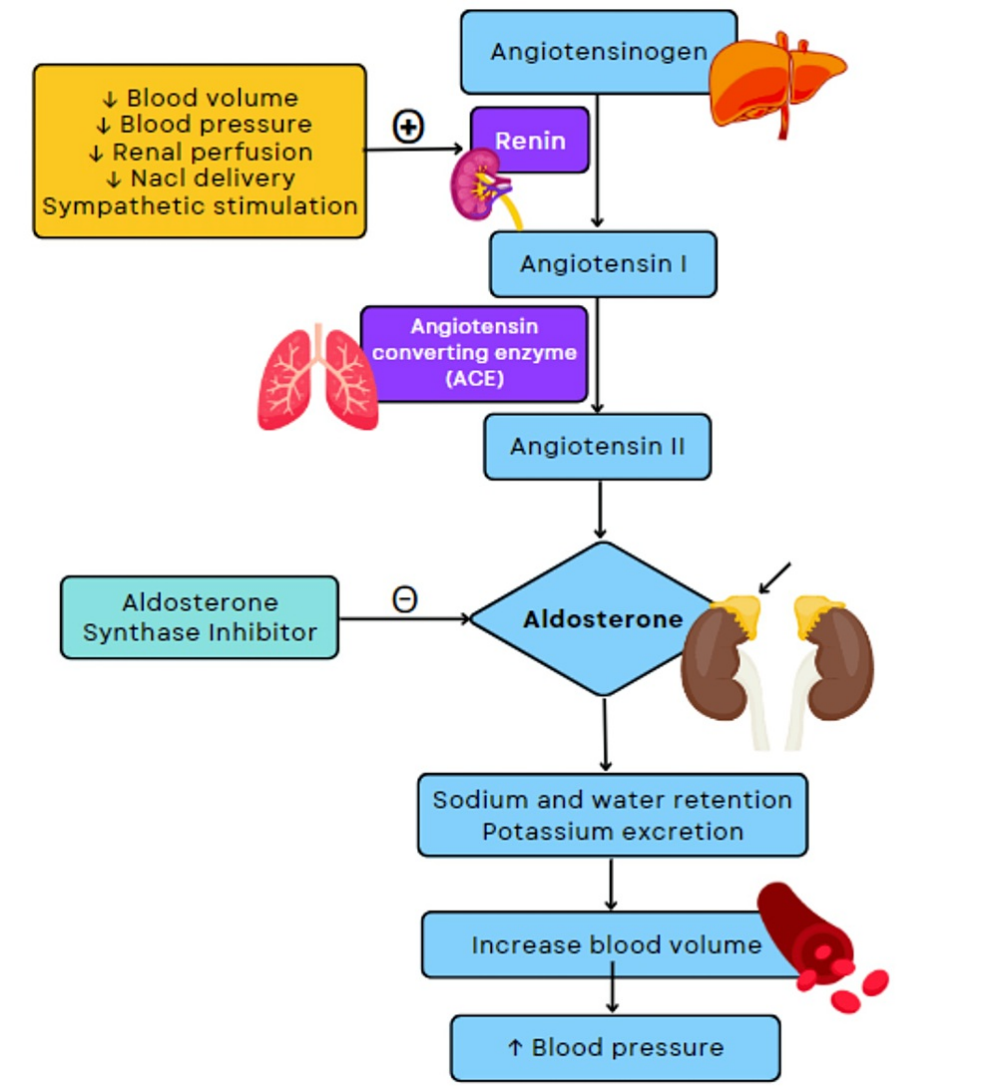
Action of aldosterone synthase inhibitor on renin-angiotensin-aldosterone system. Aldosterone synthase inhibitors decrease blood pressure by inhibiting the physiological increases in sodium and water reabsorption, blood volume, and blood pressure resulting from adequate physiological stimuli such as decreased blood volume, blood pressure, renal perfusion sodium chloride delivery, and increased sympathetic stimulation. ↓ = decrease; ↑ = increase; + = activate; - = inhibit

Aldosterone is a mineralocorticoid steroid hormone produced by the zona glomerulosa cells of the adrenal cortex [27] and is the final product obtained through the activity of the RAAS [28]. Figure 2 shows how physiological stimuli and RAAS responses increase BP.

The RAAS is responsible for producing adaptations to several conditions such as hyponatremia, hyperkalemia, hypertension, hypotension, and acid-base balance [29]. Aldosterone has a plasma half-life of less than 20 minutes and works by binding to the mineralocorticoid receptor where its primary activity involves the maintenance of normal concentrations of the plasma electrolytes sodium, potassium, bicarbonate, and hydrogen ions, as well as extracellular fluid and pH homeostasis [16,28]. If the binding capacity of aldosterone is interrupted, its plasma level is increased; such dysregulation contributes to the progression of cardiorenal diseases.

Aldosterone production is stimulated by several factors. Alteration in serum potassium is the most potent stimulator of aldosterone secretion; however, increases in plasma angiotensin II and ACTH levels also drive aldosterone synthesis and release [30]. Decreased delivery of sodium to the macula densa cells of the kidney’s distal convoluted tubules triggers the release of renin, and in turn, plasma angiotensinogen conversion to inactive angiotensin I, angiotensin I conversion to active angiotensin II and eventual release of aldosterone into the blood by the adrenal’s zona glomerulosa cells. Plasma acidosis also has a role to play in aldosterone activity, and aldosterone helps with potassium and hydrogen ion secretion for the excretion of these ions by the collecting tubules. Aldosterone is one of the regulators of sodium-potassium ATPase, also known as the sodium-potassium pump, at the plasma membrane of virtually all cells, thereby regulating the relative concentrations of sodium and potassium in the extracellular and intracellular fluids. Adrenoglomerulotropin, a lipid substance secreted by the pineal gland is also reported to stimulate aldosterone secretion [30].

Increased aldosterone activity has been shown to be associated with an increase in the risk of HTN and associated alterations in the functioning of the heart, peripheral vasculature, and kidneys [16,28]. An increase in plasma aldosterone can increase sodium reabsorption from the kidney’s filtrate regulation of epithelial sodium channel (ENaC) activity in the segment of principal cells located in the aldosterone-sensitive distal nephron. After reabsorption, sodium is transported into the extracellular fluid and bloodstream with the help of Na+/K+ ATPase present on the basolateral side, resulting in an increase in the volume of the extracellular and intravascular fluids, thereby increasing the circulating blood volume. Drugs that interfere with the secretion or action of aldosterone are in use as antihypertensives. Such antihypertensive drugs decrease BP primarily by decreasing sodium and water retention (increasing the excretion of sodium and water in urine) at the expense of increasing the retention of potassium [30].

Effects of inhibiting aldosterone activity in blood vessels

Aldosterone is associated with several pathological processes linked to blood vessels. An increase in plasma aldosterone is associated with inflammatory cell infiltration of the blood vessel endothelium and this pathological process contributes to the development and progression of arterial calcification and stiffness known as arteriosclerosis [31]. The increased plasma sodium is well content resulting from aldosterone secretion may also increase oxidative stress, which, in turn, leads to endothelial dysfunction by promoting the degradation of nitric oxide (NO), the primary endothelial-derived vascular relaxation factor that protects against hypertension. Chronic exposure to aldosterone also stimulates responses at the level of the genome, and this leads to weakened endothelial function through reduced NO synthesis and activity in healthy individuals, experimental animals, and isolated human endothelial cells [32]. Aldosterone also causes the release of endothelin-1 (ET-1) from the endothelial cells. This elicits ET receptor-mediated vasoconstriction, in addition to the enhanced vasoconstrictor tone resulting from the decreased vasodilatory activity of NO.

Mineralocorticoid receptor inhibitors, ET(A) receptor antagonists, and T-type calcium channel blockers appear to attenuate the pathologic contribution of aldosterone in vascular disease and exert valuable actions on the bioavailability of endothelium-derived NO, particularly in resistant hypertension and hyperaldosteronism. Aldosterone excess appears to be linked to arterial blood vessel dysfunction in patients diagnosed with hyperaldosteronism [33].

Effects of aldosterone on the heart

In addition to its important mineralocorticoid role, aldosterone mediates several cardiovascular processes. For example, aldosterone excess is linked to the development and progression of end-organ damage associated with hypertension and cardiac hypertrophy [31]. Hyperaldosteronism is significantly related to increased intravascular volumes, intracardiac volumes, and left ventricular mass [34]. High plasma aldosterone is also thought to be associated with cardiac inflammation and myocardial fibrosis [31,34]. These observations appear to establish excess aldosterone secretion as an important mediator of risk for developing cardiovascular disease. Animal studies on hyperaldosteronism have shown that the negative target-organ effects of aldosterone are dependent on high dietary sodium intake [34]. These findings suggest that the harmful tissue effects of aldosterone may be principally avoided by maintaining a low-salt diet. Thus, inhibition of aldosterone synthesis, either directly or indirectly via the RAAS pathway, could be a potential game-changer in reducing or preventing cardiovascular disease and related complications.

In a study of 182 never-treated patients diagnosed with primary HTN, three years of treatment with an angiotensin-converting enzyme inhibitor, an angiotensin receptor blocker (ARB), and/or other antihypertensive agents as needed for BP control, biochemical and echocardiographic evaluations showed significant reductions in left ventricular mass index which were correlated with the decrements in plasma aldosterone and SBP, and increments in urinary sodium excretion [34]. In a study of young adults, high plasma aldosterone was shown to attenuate cardiovagal and sympathetic baroreflex sensitivity, a primary pathophysiological mechanism for the development of HTN [35].

*WNK1* gene mutations are thought to cause familial hyperkalemic hypertension by increasing sodium reabsorption, blood volume, and BP. *WNK1* makes a transcription factor for synthesizing and inserting a chloride-cation transporter in the basolateral membranes of the distal nephron. The relationship between overexpression of *WNK1*, sodium retention, and HTN is not entirely clear. Two main *WNK1* transcripts have been shown to be expressed in the kidney: the formerly designated "long" WNK1 and a shorter transcript that is precisely expressed in the kidney (KS-WNK1) [36]. Aldosterone is reported to induce KS-WNK1 expression but not expression of long-WNK1 [37]. Stable overexpression of KS-WNK1 significantly increases transepithelial sodium transport in cortical collecting duct cells, and these observations suggest that stimulation of KS-WNK1 expression might be an important element of aldosterone-induced sodium retention and HTN [37].

Aldosterone and baroreceptor sensitivity

Pressure-sensitive receptors (baroreceptors) are located within the blood vessel walls of the large arteries in the thoracic cavity and neck and are particularly plentiful in the carotid and aortic sinuses. These baroreceptors are sensitive to arterial BP changes, which can affect aldosterone secretion [35]. A decrease in sensed pressure results in a decreased rate of firing by the baroreceptors, and a feedback response that increases systemic arterial pressure by increasing sympathetic activity to the heart and blood vessels with corresponding increases in blood volume [38]. Aldosterone release causes sodium and water retention in the kidney which increases blood volume and BP. Decreased BP detected by the stretch receptors located in the atria of the heart also stimulates the adrenal gland to release aldosterone to help return BP to normal [35].

One of the more interesting aspects of the aldosterone story is the baroreceptor resetting to a higher BP and a critical pathophysiological feature of treatment-resistant HTN. Sympathetic neural stimulation is shown to inhibit the activity of baroreceptors during aerobic exercise, thereby permitting the physiological increase in BP during physical activity. Aldosterone is shown to produce similar sympathetic excitation which inhibits baroreceptor activity and may, therefore, play a critical role in inhibiting baroreceptor sensitivity, required for resetting baroreceptor function to a higher-than-normal BP [33]. 

**Figure 3:**
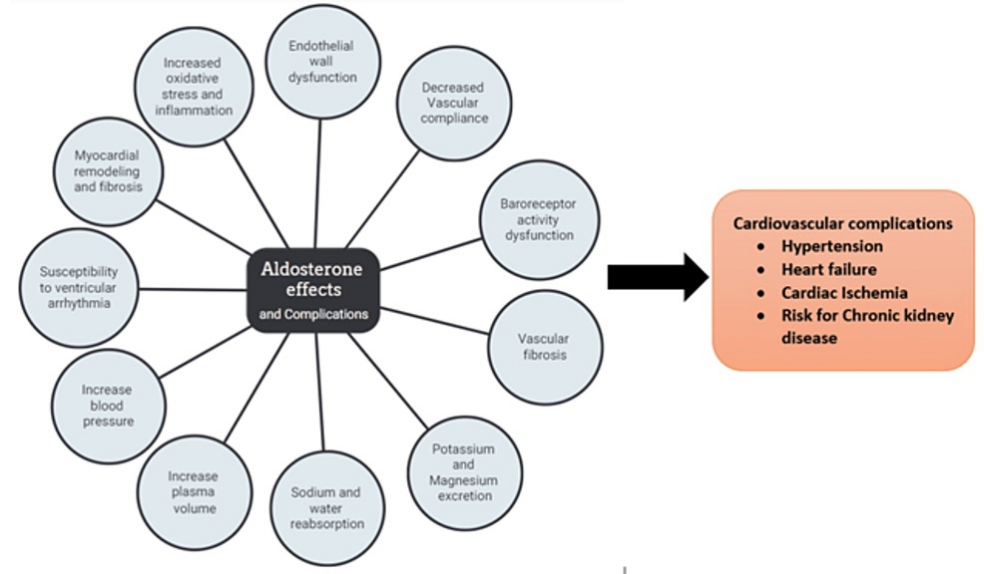
Physiological actions of aldosterone implicated in cardiovascular and related diseases.

Figure 3 summarizes the physiological actions of aldosterone that are implicated in the development and maintenance of cardiovascular and related diseases.

Novel approaches to reducing morbidity and mortality from CVD by combining dietary changes with aldosterone inhibition strategies

Salt-restricted Diets and Anti-aldosterone Synergism

The close association between high dietary sodium intake and HTN is well recognized. A large body of evidence exists showing a causal relationship between dietary sodium intake and BP [39]. The correlation between sodium intake and BP was derived from the assessment of baseline urinary sodium excretion using 24-hour urine collected by standardized methods, and BP measurement. The International Study of Sodium, Potassium, and Blood Pressure (INTERSALT) involving 10,079 adults aged 20-59 years from 32 countries, reveals a direct association between dietary sodium intake and BP [40]. These findings were validated by many other large epidemiological studies and experiments [41-44]. Experiments involving whole-of-population salt reduction have been shown to lower population BP, as revealed by countries where salt intake has been reduced successfully, for example, the United Kingdom and Finland [43,44]. Various meta-analyses of randomized control trials on salt reduction reveal a significant reduction in BP, although the degree of the fall in BP varied largely due to different inclusion and exclusion criteria [45-49]. Consequently, the World Health Organization has recommended reducing salt intake to 5 g/day (equivalent to 2 g/day sodium), to reduce the incidence of hypertension and to improve cardiovascular outcomes [50]. A further reduction to 3 g/day is even recommended by other countries to achieve more health benefits. The United Kingdom National Institute for Health and Care Excellence has recommended a long-term population target of 3 g/day of salt intake. [51]. The United States recommended 4 g/day of salt intake for over 50% of its population including individuals> 50 years old, blacks, and those with conditions like diabetes, hypertension, and chronic kidney disease [52].

Studies have also shown the additive effects of dietary salt restriction in combination with pharmacological and nonpharmacological antihypertension strategies for lowering BP [53, 54]. The Dietary Approaches to Stop Hypertension (DASH)-Sodium trial reveals that combining lower salt intake with the DASH diet (rich in fruits, vegetables, and low-fat dairy foods) results in a significantly lower BP compared to those on the usual American diet at any given salt intake level. The greatest reduction in BP was realized when the DASH diet was combined with the lowest salt intake level [55,56].

The Trial of Nonpharmacological Interventions in the Elderly (TONE) study demonstrates that following the withdrawal of antihypertensive therapy in elderly, obese, and hypertensive persons, salt reduction in combination with weight loss was more effective than either intervention alone in reducing and maintaining healthy BP [57].

A randomized double-blind trial in persons who are hypertensive on the angiotensin-converting enzyme inhibitor and RAAS blocker captopril shows that reducing salt intake by 5.8 g/day over a month lowered BP further, by 13/9 mmHg [58]. This study further highlights the addictive effect of low salt intake when combined with antihypertensive therapy.

The foregoing studies show that dietary salt restriction is associated with lower BP and switching from a “Western” to a “Mediterranean” or “Ancient” diet reduces the risk factors for developing hypertension and cardiovascular disease. However, ancient diets were low in sodium and high in potassium and the high potassium appears to make an important contribution to the health benefit by inhibiting the sodium chloride transporter in the distal nephron. Evidence is emerging that the local potassium level in the distal nephron reciprocally regulates the sodium-chloride cotransporter and epithelial sodium channel activity for adjusting sodium and potassium excretion to their dietary intakes [59]. The rationale for the dietary substitution of potassium chloride for sodium chloride, recommended by the World Health Organization, is supported by findings that potassium inhibits the sodium-chloride cotransporter in the distal convoluted tubule of the nephron [60]. However, a concurrent increase in potassium is also reported, in some situations, to increase plasma aldosterone, thereby increasing sodium retention, blood volume, and BP [60].

Low sodium diets, therefore, may have had limited success in controlling BP because low sodium intake does result in concomitant upregulation of the RAAS, aldosterone secretion, sodium, and water retention [27, 30]. The combined effect of these changes is to move BP back toward a higher BP, dictated by resetting the baroreceptors to a higher-than-normal BP [35]. Theoretically, combining a low sodium diet with an ASI should alleviate the effect of RAAS upregulation and baroreceptor resetting to a higher-than-normal BP. Aldosterone synthesis inhibition should also decrease oxidative stress and endothelial dysfunction associated with higher-than-normal BP, causing more NO synthesis, release, and vasorelaxation.

Hypertension in Salt-Sensitive Versus Salt-Insensitive Patients

Some individuals can effectively excrete salt, even after high dietary salt consumption, and they have no risk of high BP. These individuals are considered “salt-insensitive.” However, others who cannot effectively excrete often develop hypertension after high salt consumption. These individuals are called “salt-sensitive” [37]. Salt sensitivity, which is an increase in BP because of high dietary salt intake, is an independent and important risk factor for HTN and CVD progression and mortality [37]. It is associated with physiological, environmental, demographic, and genetic factors. HTN can sometimes be classified as salt-sensitive and salt-insensitive conditions depending on the patient. Persons with salt-sensitive HTN have a greater increase in BP than those with salt-insensitive HTN [53]. Even when their BP is normal, salt-sensitive individuals tend to have increased mortality from complications related to HTN, such as myocardial infarction and stroke [37]. Persons may, therefore, differ from each other regarding the development of HTN after dietary salt consumption.

**Figure 4:**
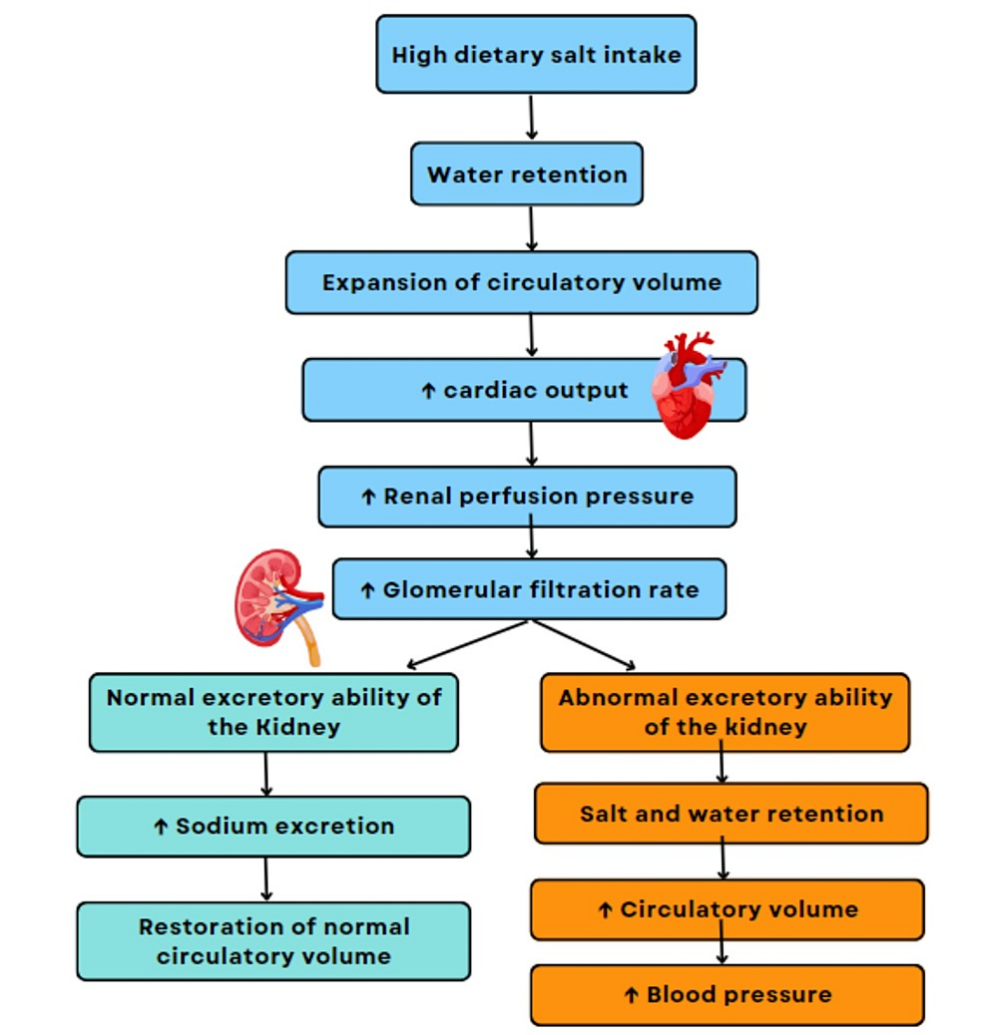
The pressure-natriuresis mechanism of blood pressure control.

It has been estimated that 26 out of 100 people with normal BP have salt sensitivity and half of hypertensive patients are thought to also be salt-sensitive [61]. This means that the BP in a salt-sensitive person often increases dramatically in response to dietary salt. Individuals who are salt-sensitive, particularly from the black population, are reported to possess a suppressed RAAS that may respond to high dietary salt with the inability to excrete enough sodium which can result in HTN. In salt-sensitive individuals, this increase in RAAS increases plasma aldosterone even when salt consumption is adjusted to the same level as for salt-insensitive individuals. Age is also shown to cause salt sensitivity in association with inadequately suppressed RAAS and secretion of aldosterone [62]. Figure 4 depicts the pressure-natriuresis mechanism by which the pressure-natriuresis mechanism of the kidney augments RAAS inhibition to increase sodium excretion in response to high dietary salt intake and protect against elevation of BP and HTN, implicating an abnormality in this mechanism in the development of salt-sensitive HTN.

Salt sensitivity has been shown to be due to an interplay of mechanisms such as RAAS, endothelial dysfunction, ion transport, and estrogen deficiency in women [63]. Higher dietary intakes of calcium, vitamin D, potassium, antioxidant vitamins, and proteins rich in L-arginine, as well as adherence to dietary options like the DASH diet, are shown to be beneficial to salt-sensitive populations. In contrast, the Western diet, which is rich in saturated fats, sucrose, and fructose, together with excessive alcohol consumption, appears to aggravate salt-sensitive fluctuations in BP [37,63]. Identifying the potential mechanisms of salt sensitivity in vulnerable populations and connecting them to protective or beneficial dietary, lifestyle factors, and cardiac rehabilitation exercises can lead to more precise strategies for the prevention of HTN and related complications such as CVD [62,64]. According to current guidelines from the American Heart Association and the American College of Cardiology Foundation, lifestyle modifications are considered a class 1b recommendation for individuals at high to moderate risk for developing CVD [65]. Table 3 summarizes the therapeutic profile of ASIs and how they could be a potential game changer in treating different types of HTN and CVD.

**Table 3:** Therapeutic summary of aldosterone synthase inhibitors ASI: aldosterone synthase inhibitor; ACTH: adrenocorticotrophic hormone; HTN: hypertension

Key takeaway points	1. ASIs are a promising and emerging class of drugs, highly effective and potent inhibitors of aldosterone synthase.
	2. Single daily dose is proven to be safe and efficient for both essential and resistant HTN.
	3. Pharmacokinetic profile: shows rapid absorption, peak plasma concentration of about 4 hours, and mean half-life ranging between 6 – 31 hours. No significant evidence of nephro- or hepatotoxicity.
	4. The pharmacodynamic profile: shows a dose-dependent decrease in plasma aldosterone level.
	5. Modest safety profile.
	6. Lesser adverse effects unlike mineralocorticoid receptor antagonists (MRAs) such as Spironolactone and Eplerenone.
	7. Baxdrostat has no significant effect on the cortisol-ACTH pathway. It blocks the 'non-genomic' effects of aldosterone
	8. Combination with a low salt diet further proves its efficacy by alleviating the negative feedback on the RAAS system when compared to a low salt diet only measure of B.P control.

## Conclusions

The potential for ASIs such as baxdrostat to safely and effectively reduce BP compared to placebo in patients diagnosed with CVD has been sufficiently proven. Baxdrostat was effective in reducing plasma aldosterone in the presence and absence (normal dietary salt) of dietary salt restriction. The 2.5 mg dose of baxdrostat was found to be equally effective in lowering aldosterone levels in both situations. Baxdrostat appears to suppress aldosterone secretion without having any significant impact on cortisol secretion, even when the administration of baxdrostat is associated with an ACTH provocation test to stimulate the synthesis of cortisol and aldosterone. This finding further demonstrates the selectivity of baxdrostat in targeting AS. Compared to placebo, baxdrostat also significantly reduced BP in treatment-resistant hypertensive patients. However, these advantages will need to be confirmed in phase 3 trials involving more patients over a longer period and in comparison, to other anti-hypertensive drugs as well.

Baxdrostat seems to provide a new powerful tool for patients with resistant HTN who may be able to reduce their risk of cerebrovascular accidents as well as the overall morbidity and mortality from CVD if phase 3 trials show encouraging results. Additionally, it is suggested that individuals who are taking medication for high BP also incorporate lifestyle changes as an adjunctive treatment. Weight loss, vigorous aerobic activity in fitness facilities or cardiac rehabilitation centers, and the DASH diet with sodium restriction will all help high-risk persons lower their overall risk for morbidity and mortality associated with HTN and CVD even further.

## References

[1] Williams GH (1994). Essential hypertension as an endocrine disease. Endocrinol Metab Clin North Am.

[2] Grossmann C, Gekle M (2009). New aspects of rapid aldosterone signaling. Mol Cell Endocrinol.

[3] Good DW (2007). Nongenomic actions of aldosterone on the renal tubule. Hypertension.

[4] Mihailidou AS, Funder JW (2005). Nongenomic effects of mineralocorticoid receptor activation in the cardiovascular system. Steroids.

[5] Calhoun DA, White WB, Krum H (2011). Effects of a novel aldosterone synthase inhibitor for treatment of primary hypertension: results of a randomized, double-blind, placebo- and active-controlled phase 2 trial. Circulation.

[6] Guyton AC (1991). Blood pressure control--special role of the kidneys and body fluids. Science.

[7] Girardin E, Caverzasio J, Iwai J, Bonjour JP, Muller AF, Grandchamp A (1980). Pressure natriuresis in isolated kidneys from hypertension-prone and hypertension-resistant rats (Dahl rats). Kidney Int.

[8] MacGregor GA, Markandu ND, Roulston JE, Jones JC, Morton JJ (1981). Maintenance of blood pressure by the renin-angiotensin system in normal man. Nature.

[9] Yoon HE, Choi BS (2014). The renin-angiotensin system and aging in the kidney. Korean J Intern Med.

[10] Fattah H, Layton A, Vallon V (2019). How do kidneys adapt to a deficit or loss in nephron number?. Physiology (Bethesda).

[11] Freeman MW, Bond M, Murphy B, Hui J, Isaacsohn J (2023). Results from a phase 1, randomized, double-blind, multiple ascending dose study characterizing the pharmacokinetics and demonstrating the safety and selectivity of the aldosterone synthase inhibitor baxdrostat in healthy volunteers. Hypertens Res.

[12] Freeman MW, Halvorsen YD, Marshall W (2023). Phase 2 trial of baxdrostat for treatment-resistant hypertension. N Engl J Med.

[13] Sloan-Lancaster J, Raddad E, Flynt A, Jin Y, Voelker J, Miller JW (2017). LY3045697: Results from two randomized clinical trials of a novel inhibitor of aldosterone synthase. J Renin Angiotensin Aldosterone Syst.

[14] Ménard J, Rigel DF, Watson C (2014). Aldosterone synthase inhibition: cardiorenal protection in animal disease models and translation of hormonal effects to human subjects. J Transl Med.

[15] Schumacher CD, Steele RE, Brunner HR (2013). Aldosterone synthase inhibition for the treatment of hypertension and the derived mechanistic requirements for a new therapeutic strategy. J Hypertens.

[16] Karns AD, Bral JM, Hartman D, Peppard T, Schumacher C (2013). Study of aldosterone synthase inhibition as an add-on therapy in resistant hypertension. J Clin Hypertens (Greenwich).

[17] Andersen K, Hartman D, Peppard T, Hermann D, Van Ess P, Lefkowitz M, Trapani A (2012). The effects of aldosterone synthase inhibition on aldosterone and cortisol in patients with hypertension: a phase II, randomized, double-blind, placebo-controlled, multicenter study. J Clin Hypertens (Greenwich).

[18] Taves MD, Gomez-Sanchez CE, Soma KK (2011). Extra-adrenal glucocorticoids and mineralocorticoids: evidence for local synthesis, regulation, and function. Am J Physiol Endocrinol Metab.

[19] Silvestre JS, Heymes C, Oubénaïssa A (1999). Activation of cardiac aldosterone production in rat myocardial infarction: effect of angiotensin II receptor blockade and role in cardiac fibrosis. Circulation.

[20] White PC (2004). Aldosterone synthase deficiency and related disorders. Mol Cell Endocrinol.

[21] Veldhuis JD, Melby JC (1981). Isolated aldosterone deficiency in man: acquired and inborn errors in the biosynthesis or action of aldosterone. Endocr Rev.

[22] Chapman N, Dobson J, Wilson S, Dahlöf B, Sever PS, Wedel H, Poulter NR (2007). Effect of spironolactone on blood pressure in subjects with resistant hypertension. Hypertension.

[23] Calhoun DA, White WB (2008). Effectiveness of the selective aldosterone blocker, eplerenone, in patients with resistant hypertension. J Am Soc Hypertens.

[24] Forzano I, Mone P, Varzideh F, Jankauskas SS, Kansakar U, De Luca A, Santulli G (2022). The selective aldosterone synthase inhibitor baxdrostat significantly lowers blood pressure in patients with resistant hypertension. Front Endocrinol (Lausanne).

[25] Williams B, MacDonald TM, Morant SV (2018). Endocrine and haemodynamic changes in resistant hypertension, and blood pressure responses to spironolactone or amiloride: the PATHWAY-2 mechanisms substudies. Lancet Diabetes Endocrinol.

[26] Williams B, MacDonald TM, Morant S (2015). Spironolactone versus placebo, bisoprolol, and doxazosin to determine the optimal treatment for drug-resistant hypertension (PATHWAY-2): a randomised, double-blind, crossover trial. Lancet.

[27] Tait SA, Tait JF, Coghlan JP (2004). The discovery, isolation and identification of aldosterone: reflections on emerging regulation and function. Mol Cell Endocrinol.

[28] Wang H, Tian J, Yang K (2015). Efficacy and safety of LCI699 for hypertension: a meta-analysis of randomized controlled trials and systematic review. Eur Rev Med Pharmacol Sci.

[29] Millis RM (2009). Epigenetics, essential hypertension and renin-angiotensin system upregulation in the offspring of water-deprived pregnant rats. Hypertens Res.

[30] Tsilosani A, Gao C, Zhang W (2022). Aldosterone-regulated sodium transport and blood pressure. Front Physiol.

[31] Buffolo F, Tetti M, Mulatero P, Monticone S (2022). Aldosterone as a mediator of cardiovascular damage. Hypertension.

[32] Toda N, Nakanishi S, Tanabe S (2013). Aldosterone affects blood flow and vascular tone regulated by endothelium-derived NO: therapeutic implications. Br J Pharmacol.

[33] Hung CS, Sung SH, Liao CW (2019). Aldosterone induces vascular damage. Hypertension.

[34] Szczepanska-Sadowska E (2022). The heart as a target of vasopressin and other cardiovascular peptides in health and cardiovascular diseases. Int J Mol Sci.

[35] Acelajado MC, Pimenta E, Calhoun DA (2010). Salt and aldosterone: a concert of bad effects. Hypertension.

[36] Monahan KD, Leuenberger UA, Ray CA (2007). Aldosterone impairs baroreflex sensitivity in healthy adults. Am J Physiol Heart Circ Physiol.

[37] Náray-Fejes-Tóth A, Snyder PM, Fejes-Tóth G (2004). The kidney-specific WNK1 isoform is induced by aldosterone and stimulates epithelial sodium channel-mediated Na+ transport. Proc Natl Acad Sci U S A.

[38] Awosika A, Adabanya U, Millis RM, Omole AE, Moon JH (2023). Postprandial hypotension: an underreported silent killer in the aged. Cureus.

[39] Grobbee DE, Hofman A (1986). Does sodium restriction lower blood pressure?. Br Med J (Clin Res Ed).

[40] (1988). Intersalt: an international study of electrolyte excretion and blood pressure. Results for 24 hour urinary sodium and potassium excretion. Intersalt Cooperative Research Group. BMJ.

[41] Zhou BF, Stamler J, Dennis B (2003). Nutrient intakes of middle-aged men and women in China, Japan, United Kingdom, and United States in the late 1990s: the INTERMAP study. J Hum Hypertens.

[42] Khaw KT, Bingham S, Welch A, Luben R, O'Brien E, Wareham N, Day N (2004). Blood pressure and urinary sodium in men and women: the Norfolk Cohort of the European Prospective Investigation into Cancer (EPIC-Norfolk). Am J Clin Nutr.

[43] He FJ, Pombo-Rodrigues S, Macgregor GA (2014). Salt reduction in England from 2003 to 2011: its relationship to blood pressure, stroke and ischaemic heart disease mortality. BMJ Open.

[44] Karppanen H, Mervaala E (2006). Sodium intake and hypertension. Prog Cardiovasc Dis.

[45] Law MR, Frost CD, Wald NJ (1991). By how much does dietary salt reduction lower blood pressure? III--analysis of data from trials of salt reduction. BMJ.

[46] Geleijnse JM, Kok FJ, Grobbee DE (2003). Blood pressure response to changes in sodium and potassium intake: a metaregression analysis of randomised trials. J Hum Hypertens.

[47] Midgley JP, Matthew AG, Greenwood CM (1996). Effect of reduced dietary sodium on blood pressure: a meta-analysis of randomized controlled trials. JAMA.

[48] Graudal NA, Galløe AM, Garred P (1998). Effects of sodium restriction on blood pressure, renin, aldosterone, catecholamines, cholesterols, and triglyceride: a meta-analysis. JAMA.

[49] Aburto NJ, Ziolkovska A, Hooper L, Elliott P, Cappuccio FP, Meerpohl JJ (2013). Effect of lower sodium intake on health: systematic review and meta-analyses. BMJ.

[50] (2012). World Health Organization. Guideline: Sodium Intake for Adults and Children; World Health Organization: Geneva, Switzerland, 2012. Guideline: Sodium Intake for Adults and Children.

[51] (2010). Effect of lower sodium intake on health: systematic review and meta-analyses. Cardiovascular Disease Prevention.

[52] (2015). U.S. Department of Health and Human Services and U.S. Department of Agriculture. 2015 - 2020 Dietary Guidelines for Americans. Dietary Guidelines for Americans 2015 - 2020, Eighth Edition.

[53] Choi HY, Park HC, Ha SK (2015). Salt sensitivity and hypertension: a paradigm shift from kidney malfunction to vascular endothelial dysfunction. Electrolyte Blood Press.

[54] Thout SR, Santos JA, McKenzie B (2019). The science of salt: updating the evidence on global estimates of salt intake. J Clin Hypertens (Greenwich).

[55] He FJ, MacGregor GA (2010). Reducing population salt intake worldwide: from evidence to implementation. Prog Cardiovasc Dis.

[56] Sacks FM, Svetkey LP, Vollmer WM (2001). Effects on blood pressure of reduced dietary sodium and the dietary approaches to stop hypertension (DASH) diet. DASH-sodium collaborative research group. N Engl J Med.

[57] Juraschek SP, Miller ER 3rd, Weaver CM, Appel LJ (2017). Effects of sodium reduction and the DASH diet in relation to baseline blood pressure. J Am Coll Cardiol.

[58] Whelton PK, Appel LJ, Espeland MA (1998). Sodium reduction and weight loss in the treatment of hypertension in older persons: a randomized controlled trial of nonpharmacologic interventions in the elderly (TONE). TONE Collaborative Research Group. JAMA.

[59] MacGregor GA, Markandu ND, Singer DR, Cappuccio FP, Shore AC, Sagnella GA (1987). Moderate sodium restriction with angiotensin converting enzyme inhibitor in essential hypertension: a double blind study. Br Med J (Clin Res Ed).

[60] He FJ, MacGregor GA (2002). Effect of modest salt reduction on blood pressure: a meta-analysis of randomized trials. Implications for public health. J Hum Hypertens.

[61] Cornelius RJ, Wen D, Li H, Yuan Y, Wang-France J, Warner PC, Sansom SC (2015). Low Na, high K diet and the role of aldosterone in BK-mediated K excretion. PLoS One.

[62] Jürgens G, Graudal NA (2004). Effects of low sodium diet versus high sodium diet on blood pressure, renin, aldosterone, catecholamines, cholesterols, and triglyceride. Cochrane Database Syst Rev.

[63] Pilic L, Pedlar CR, Mavrommatis Y (2016). Salt-sensitive hypertension: mechanisms and effects of dietary and other lifestyle factors. Nutr Rev.

[64] Awosika A, Hillman AR, Millis RM, Adeniyi MJ (2022). Cardiac rehabilitation and cardiopulmonary fitness in children and young adults with congenital heart diseases: a critically appraised topic. Cureus.

[65] Smith SC Jr, Benjamin EJ, Bonow RO (2011). AHA/ACCF secondary prevention and risk reduction therapy for patients with coronary and other atherosclerotic vascular disease: 2011 update: a guideline from the American Heart Association and American College of Cardiology Foundation. Circulation.

